# Proximal near-infrared hyperspectral imaging dataset for identifying epicuticular wax loss in Masena blueberries to evaluate post-harvest quality

**DOI:** 10.1016/j.dib.2025.111946

**Published:** 2025-08-05

**Authors:** Shah Faisal, Yaminn Thawdar, Melanie Po-Leen Ooi, Peter Reutemann, Dale Fletcher, Ye Chow Kuang, Sanush K. Abeysekera

**Affiliations:** a School of Engineering, The University of Waikato, Hamilton 3216, New Zealand; b School of Computing and Mathematical Sciences, The University of Waikato, Hamilton 3216, New Zealand

**Keywords:** Close-range hyperspectral imaging, Smart agriculture, Blueberry, Post-harvest freshness, Non-destructive analysis

## Abstract

The dataset presented in this paper consists of hyperspectral images of Masena blueberries that were harvested on November 24, 2023, from an orchard in Pukehina, New Zealand. Blueberries were hand-harvested with gloves (intact wax), hand-harvested (without gloves), and mechanically aided by picking via a handheld shaker. Some berries were also wiped to eliminate degrading epicuticular wax (EW) for comparison. Imaging was performed within 9 hours of harvest using a Specim FX17e hyperspectral camera (900–1700 nm, 224 bands) under controlled lighting conditions. The data were white and dark reference-normalized, annotated using the in-house HAPPy tool (ENVI Software), and saved in MATLAB (.mat) format for analysis. A total of 49 individual hyperspectral images were captured from 39 blueberry fruits to capture multiple views or surface states. We provide 5 spectral hypercube sets of data collected with the hyperspectral camera: ‘Assisted Harvested Blueberries (AHB)’ (10 images), ‘Hand Harvested Blueberries (HHB)’ (10 images), ‘Perfect EW’ (10 images), ‘No EW’ (9 images), and ‘No EW vs. Perfect EW‘ (10 images: 5 from ‘No EW‘ and 5 from ‘Perfect EW‘). This dataset, collected and archived by the University of Waikato (WaI2M: Waikato Instrumentation and Measurement Research Group, Hyperspectral Imaging Group), enables near-infrared hyperspectral imaging research in agriculture for EW classification and detection, harvesting method classification, and fruit surface property spectral analysis using machine/deep learning methods.

Specifications Table

**Table:** 

Subject	*Agriculture Engineering, Hyperspectral Imaging*
Specific subject area	Assessment of post-harvest blueberries fruit quality by epicuticular wax loss and near-infrared spectral measurement.
Type of data	*MATLAB files that include 3D hypercubes, 2D Final Masks, and Lambda values.*
Data collection	*Masena blueberries with a thick Epicuticular Wax (EW) layer were harvested either manually (*i.e.*, hand-harvested, labelled HH) or mechanically (*i.e.*, Assisted-Harvested, labelled AH) in the morning in Pukehina, New Zealand. 40 fruits were randomly sampled for quality assessment and transported in an air-conditioned vehicle to the inspection site in Hamilton within 3 h in 3D-printed polylactic acid (PLA) t*rays [1]. 1 fruit *was damaged in the process and discarded. 39 fruits were imaged using a Specim FX17e camera (900–1700* nm*, 224 bands with 7* ms *exposure time, 7.21* Hz *frame rate, and 35* cm *imaging distance). In-house ENVI software (HAPP*y) [2] wa*s used to view, annotate, and convert the files to MATLAB (.mat) format.*
Data source location	*Blueberries were harvested in Pukehina, Bay of Plenty, New Zealand, and transported to the University of Waikato, Hamilton, New Zealand, for hyperspectral imaging and fruit quality assessment.*
Data accessibility	Repository name: Harvard Dataverse Data identification number: 10.7910/DVN/ENJYTC Direct URL to data: https://dataverse.harvard.edu/dataset.xhtml?persistentId=doi:10.7910/DVN/ENJYTC
Related research article	[1] J. Pearse et al., “Hyperspectral Imaging and Machine Learning to Identify Epicuticular Wax Loss in Masena Blueberries for Post-Harvest Freshness,” in 2024 IEEE 20th International Conference on Automation Science and Engineering (CASE), IEEE, Aug. 2024, pp. 2793–2798. doi: 10.1109/CASE59546.2024.10711590.

## 1 Value of the Data

• The crops imaged in this dataset are sampled for quality assessment of an industrial high-volume food production process, i.e., a commercial orchard under a controlled-field environment (tunnel-covered), making the dataset suitable for precision horticulture studies as well as post-harvest quality evaluation.
• The dataset offers high-resolution close-range hyperspectral images (3D hypercube) of blueberries harvested with different methods to analyze their effect on epicuticular wax (EW) preservation.
• This dataset enables rapid, non-destructive, and accurate identification of epicuticular wax loss, which is crucial in post-harvest freshness.
• The dataset provides annotated samples with in-house HAPPy tool support for reproducible research to construct and evaluate machine/deep learning methods for fruit surface analysis, quality evaluation, and harvesting method classification.

## 2 Background

The increasing global shift for automated agricultural technologies [3] has driven the research for preserving post-harvest quality of fruits. Specifically, Epicuticular Wax (EW), which is a substance that appears as a white, powdery layer on the surface of the fruit is a key indicator of quality [4]. In pre-harvest, EW reduces fruit susceptibility to pathogenic organisms, and the chance of deformations. In post-harvest, EW increases moisture retention, prevents microbial infections, and helps resist physical damage.

As hand-harvesting (HH) becomes increasingly replaced with automation i.e. Assisted Harvesting (AH), the EW layer is affected but is difficult to assess without consistent and reliable methods. Visual observation is slow, and RGB imaging is susceptible to lighting [5]. Near-infrared hyperspectral imaging (HSI) captures spectral signatures of EW with higher consistency, thus facilitating a reliable high-volume fruit quality assessment process. This reduces the risk of throwing away good fruits (lowering profits) or retaining lower-quality fruit (potentially harming long-term reputation).

The HSI dataset was acquired as a subset of a wider project investigating the cost-benefits of replacing manual labour with mechanical harvesters. The data is complementary to another related article [1] by enabling replication of spectral-spatial analysis methods and facilitating further research work in precision agriculture. To the best of our knowledge, this is the first publicly available proximal near-infrared HSI dataset focused on evaluating epicuticular wax degradation in blueberries as a post-harvest quality indicator. Unlike existing HSI datasets, which focus largely on disease classification or remote sensing applications [6], this dataset addresses a highly specific and underexplored problem in the automation of fruit post-harvesting quality evaluation. Furthermore, because of the high acquisition costs, proprietary constraints, and competitive research objectives, data is rarely shared in the field of proximal HSI for post-harvest fruit quality.

## 3 Data Description

The data is organized under a top-level directory called **“Blueberry_HSI_Dataset”** containing five MATLAB (.mat) files. Each file is a distinct category of hyperspectral image data for blueberry fruits harvested or stored in different conditions of wax. There are 49 unique hyperspectral images collected from 39 distinct individual berry fruits. These images were arranged under five experimental groups: (i) Assisted Harvested Blueberries (AHB), (ii) Hand Harvested Blueberries (HHB), (iii) Bloom (Perfect Epicuticular Wax), (iv) No Bloom (No Wax), and (v) Combination of Bloom and No Bloom. All the files are in (.mat) format, which is suitable for hyperspectral data analysis by MATLAB, Python, or other compatible tools.

*Each .mat file contains the following DataArrays:*

• **normcube:** a 3D hyperspectral hypercube, size 1046 × 640 × 224, whose first two axes are spatial resolution and whose third axis is 224 spectral bands.
• **FinalMask:** a 2D label mask of dimensions (1046 × 640) for region-of-interest extraction and recognition.
• **lambda:** a 224 × 7 matrix of spectral wavelength values corresponding to each band.

Table 1 provides a detailed overview of all the data files, including the image number and dimensional attributes.

**Table 1:** Summary of hyperspectral image files in the “Blueberry_HSI_Dataset” folder.

Category	Name	Images	3D Hypercube Dimensions	2D Final Mask Dimensions	Lambda Dimensions
Assisted Harvested Blueberries (AHB)	10AHB_2023–11–24_00–54–21.1.mat	10	1046×640×224	1046×640	24×7
Hand Harvested Blueberries (HHB)	10HPB_2023–11–24_00–45–33.1	10	1046×640×224	1046×640	24×7
Bloom	10PerfectBloomB_2023–11–24_01–09–09.1.mat	10	1046×640×224	1046×640	24×7
No Bloom	9NoBloomB_2023–11–24_01–23–14.1.mat	9	1046×640×224	1046×640	24×7
BloomVsNoBloom	10BloomVsNoBloomB_2023–11–24_01–33–21.1.mat	10	1046×640×224	1046×640	24×7
**Total**		**49**			

The FinalMask uses a fixed label scheme across all categories for pixel extraction and classification: label 0 indicates unlabelled regions, label 1 corresponds to the background, label 2 to Bloom regions, label 3 to No Bloom regions, and label 4 to the white reference area. The description of the mask labels used by FinalMask in each file is given in Table 2. This labeling allows for robust region-based extraction and analysis across different experimental conditions.

**Table 2:** Description of mask labels used in FinalMask for each file.

Label	Meaning	Categories Where Used
0	Unlabeled	All
1	Background	All
2	Bloom	AHB, HHB, Bloom, BloomVsNoBloom
3	No Bloom	NoBloom, BloomVsNoBloom
4	White Reference	All

The names of the files follow a naming convention:

***<Number><CategoryAbbreviation>_<YYYY-MM-DD>_<HH-MM-SS.S>.mat***, in which 〈Number〉 is the quantity of hyperspectral images within the file, 〈CategoryAbbreviation〉 is the category of sample (e.g., AHB, HPB, PerfectBloomB), and the date and time are the acquisition session. For example, ***"10AHB_2023–11–24_00–54–21.1.mat"*** indicates a file containing 10 images of assisted harvested blueberries collected on November 24, 2023, at 00:54:21.1.

**Fig. 1:**
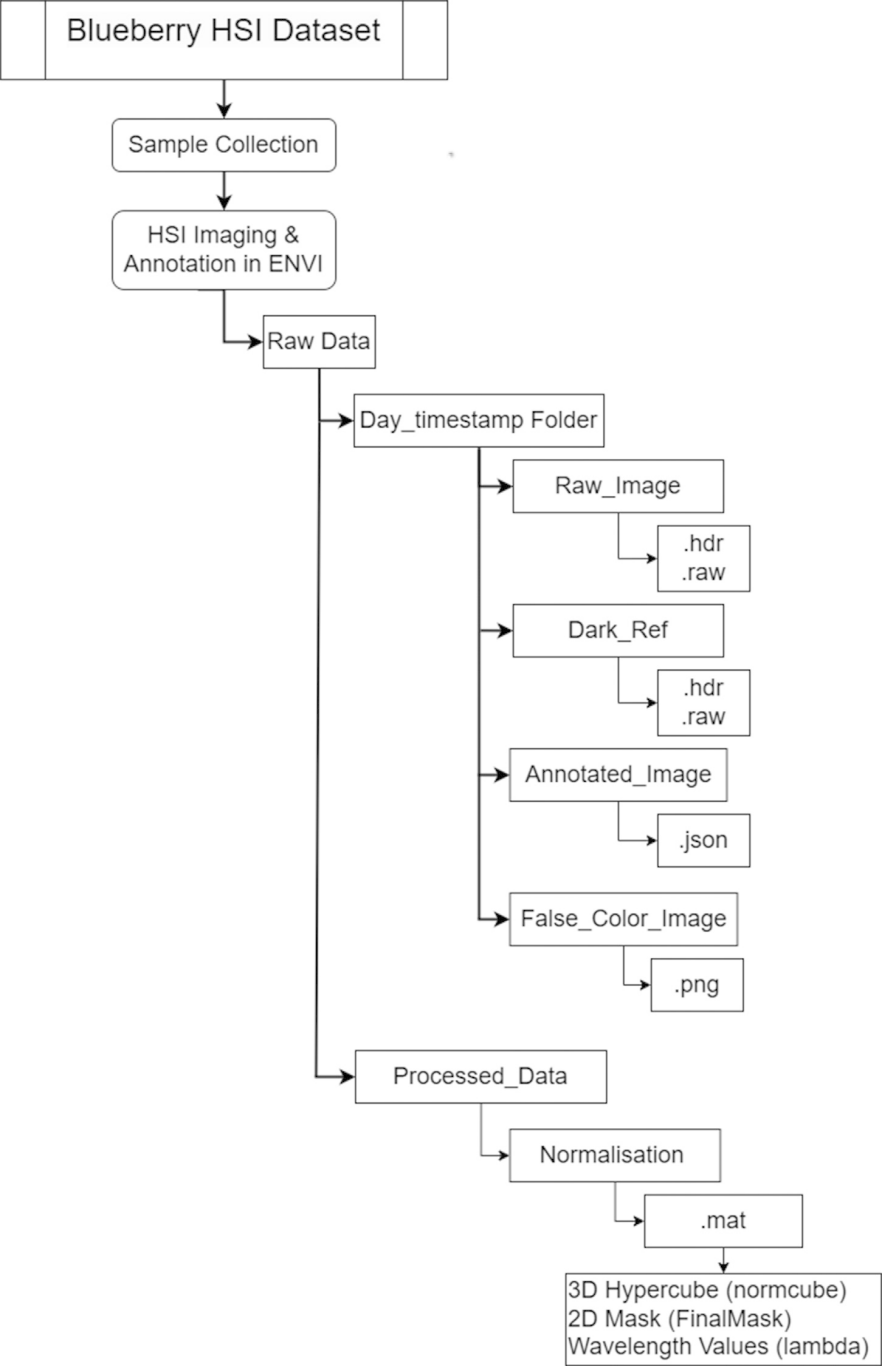
Blueberry HSI dataset processing pipeline.

Fig. 1. illustrates the blueberry hyperspectral dataset end-to-end processing pipeline, from sample collection, raw hyperspectral image acquisition (.hdr/.raw/png files), and ENVI-based annotation (.json) to final processed data outputs (.mat files containing normalized 3D hypercubes, 2D masks, and wavelength values). The key characteristics of the dataset are given in Table 3.

**Table 3:** Summary of dataset key characteristics.

Characteristic	Description
Spectral Range	*970–1700* nm *(near-infrared range)*
Spectral Dimensions	*224 bands*
Spatial Dimensions	*640 (width) × 1046 (height) pixels*
Number of Spectral Bands	*224 bands (Initially) and 230 bands (after preprocessing)*
Annotated ROI Classes	*5 classes: Unlabeled, Background, EW, No EW, White Reference*
Sampling Method & Diversity	*Random selection from a single orchard (cultivar: Masena)*
Number of Spectral Cubes	*5 sets: No EW, Perfect EW, No EW* vs *Perfect EW, AHB, HHB*
File Format	*MATLAB Files (.mat files containing normalized 3D hypercubes, 2D masks, and wavelength values).*

**Fig. 2:**
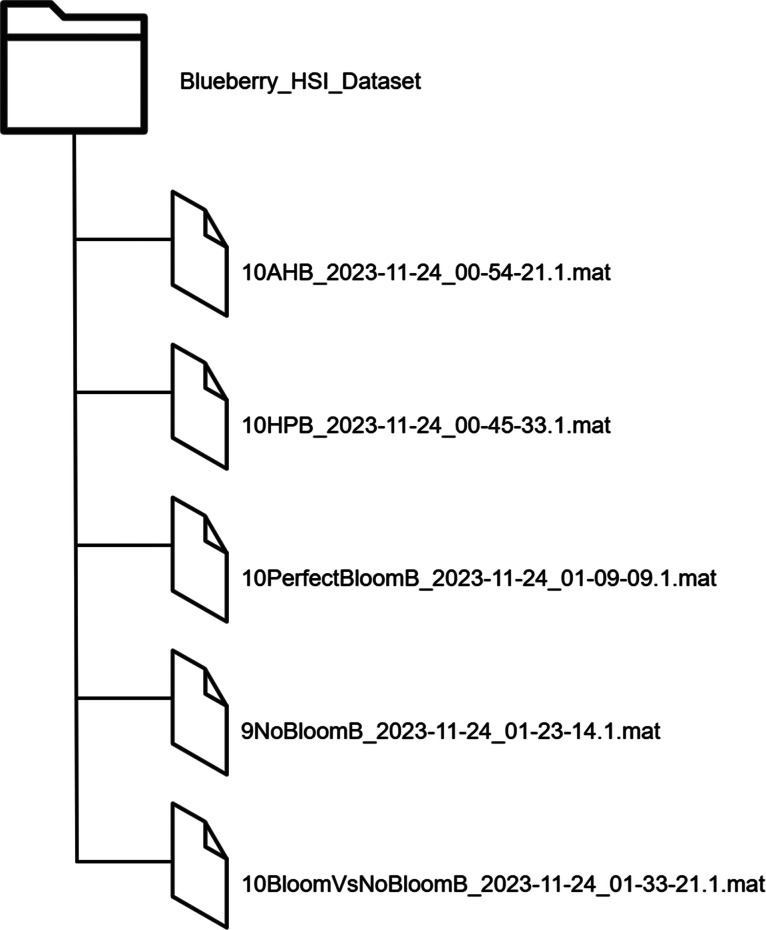
Schematic overview of the “Blueberry_HSI_Dataset” folder.

Fig. 2. presents a schematic overview of the Blueberry_HSI_Dataset folder structure and contents of the dataset.

## 4 Experimental Design, Materials and Methods

Three main stages were employed to obtain the dataset: i) sample collection, ii) hyperspectral imaging setup, and iii) data annotation and preprocessing, as explained below.

**Fig. 3:**
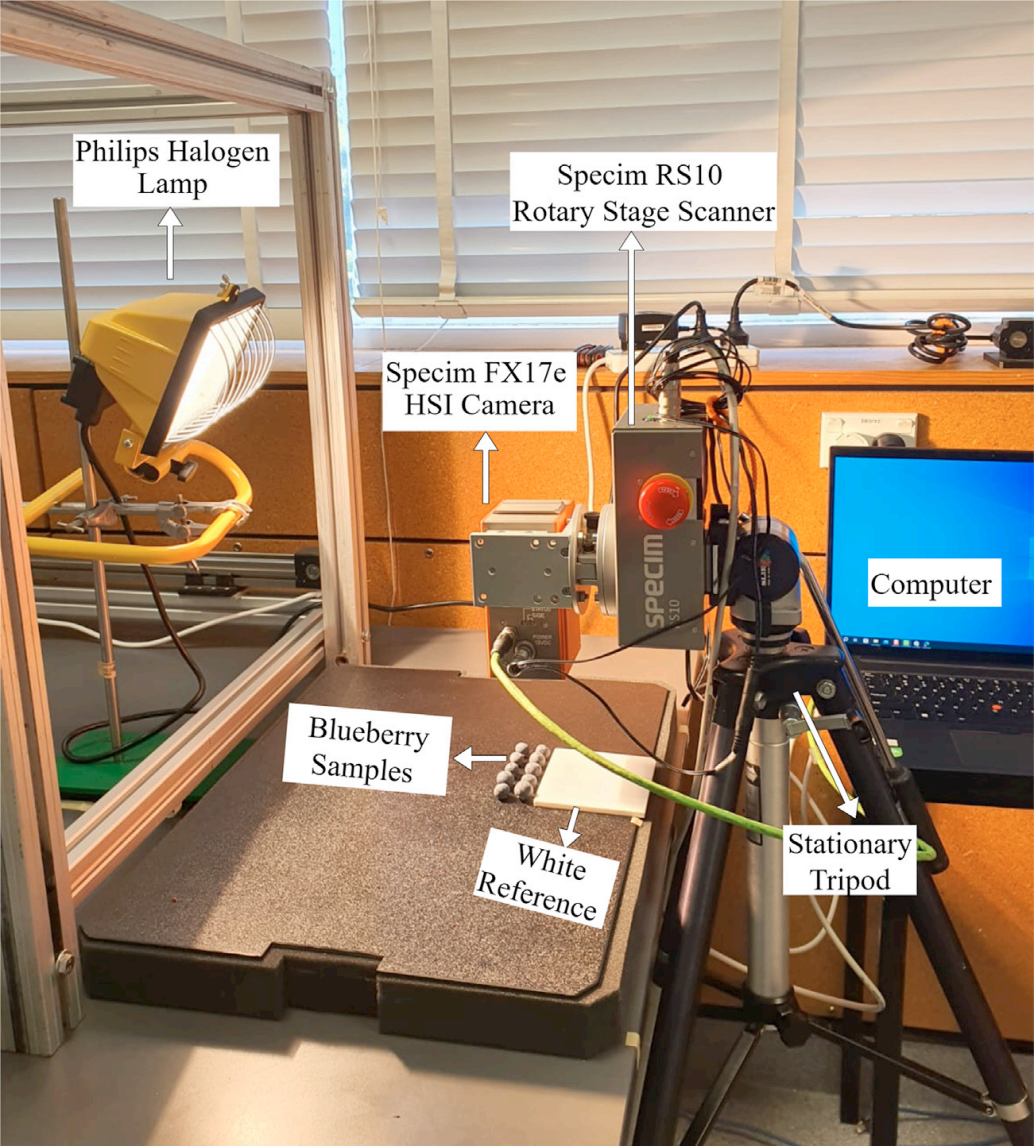
Overview of the hyperspectral imaging setup used for blueberry data collection.

**Fig. 4:**
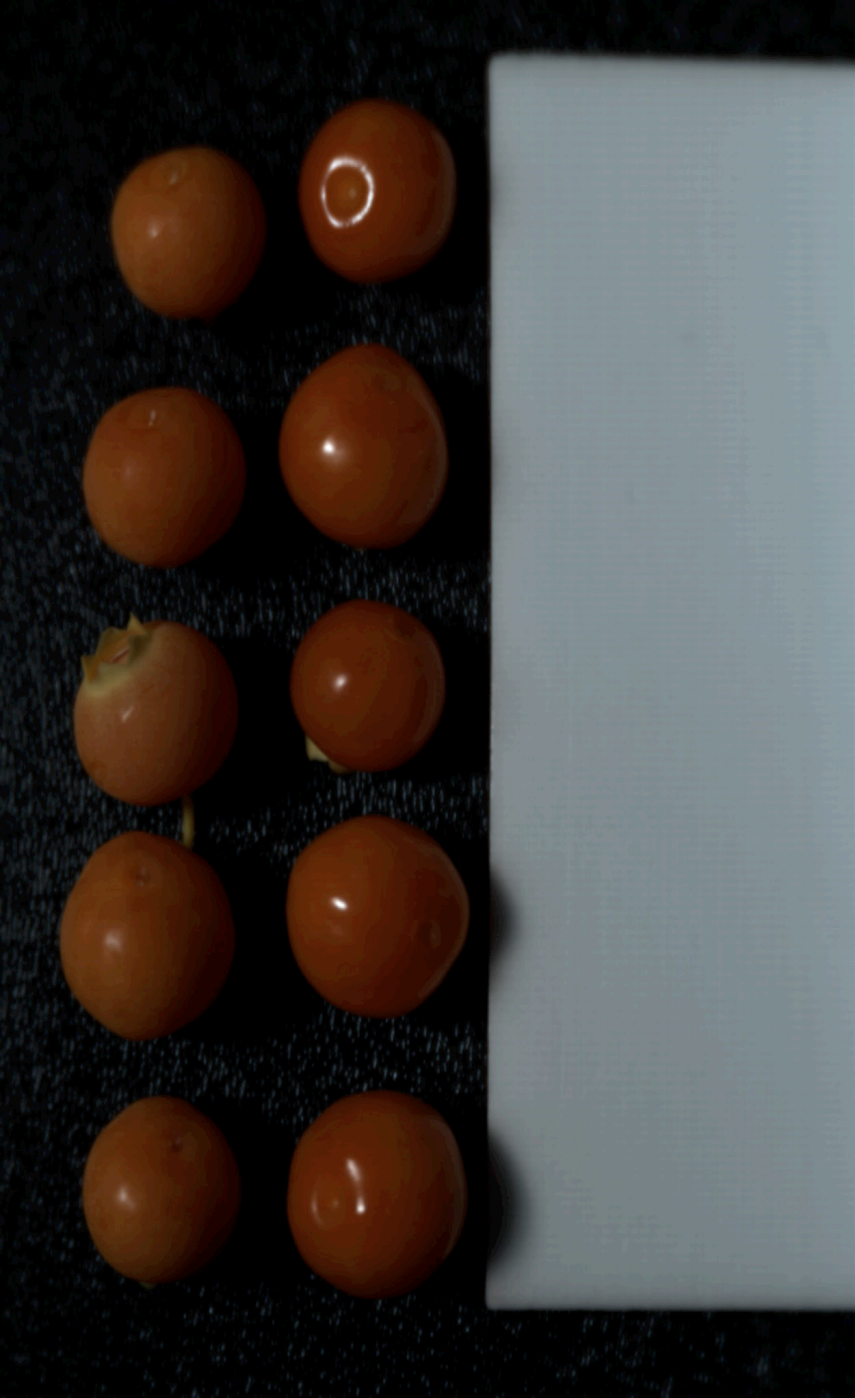
An example of a false-colour image of the blueberries under the NIR-HSI system.
• **Data Annotation and Preprocessing:**
  Image annotation was conducted using an in-house custom HAPPy tool (ENVI framework) developed by the authors [2]. Because the dataset was small, all the annotations were manually performed to ensure accuracy and control. Based on visual inspection, samples were annotated manually into five regions of interest (ROIs): (0) Unlabeled area, (1) Background, (2) EW (epicuticular wax present), (3) No EW (wax absent), and (4) White reference. An example of an annotated image is shown in Fig. 5. The labels were saved as JSON files in the Object-Predictions-Exchange (OPEX) format [11]. Raw hyperspectral data pre-processing included standard normalization with dark and white references to eliminate background noise and illumination variability and ensure consistency between imaging sessions [12]. All files were converted to .mat files to process them subsequently in MATLAB, Python, or other compatible platforms.

• **Sample Collection:**
  The dataset present for this paper was collected from ripe Masena blueberries harvested from a commercial Pukehina blueberry orchard in New Zealand. To preserve the epicuticular wax (EW) and for the purposes of uniform fruit quality, the blueberry bushes were grown under protective tunnel structures [1]. Sampling was done mid-season on November 24, 2023. Blueberries were harvested by three methods: conventional hand-picking (HH) without gloves into buckets, precision hand-picking with control sample gloves, and assisted harvesting (AH) using an improved hand-held shaker manufactured by the University of Waikato [7].
  For the control condition, 20 ripe blueberries were selected by hand while wearing polyvinyl chloride (PVC) gloves and placed into 3D-printed polylactic acid (PLA) trays. These were marked as ‘control’ with nearly perfect EW intact. 10 berries were hand-picked using traditional HH methods without gloves and transferred into a plastic bucket, then hand-picked using gloves into another PLA tray to minimize surface contact. Similarly, 10 berries were picked using the AH device and placed in another PLA tray. In the process, a single fruit was damaged and discarded. All samples were kept in an air-conditioned vehicle and driven within 3 h to the University of Waikato, where they were refrigerated and imaged within 9 h of harvesting.
• **Hyperspectral Imaging Setup:**
  Hyperspectral imaging (HSI) was conducted using a Specim FX17e camera that captures near-infrared (NIR) spectral reflectance from 900 nm to 1700 nm in 224 bands at a spatial resolution of 1046 × 640 pixels. Unlike regular RGB cameras, which capture three spectral bands, the FX17e camera captures hundreds, which enables detailed characterization of surface reflectance properties. The HSI camera was mounted on a stationary tripod and connected to a computer via a GigE M12 X-coded Ethernet cable. The camera uses a pushbroom line scanning method [8], and its movement was controlled by a rotary stage that was linked to the Specim Lumo Scanner software [9]. Sample rotation was at 5.32° per second during image capture. A Philips Plusline Halogen 500 W R7s 240 V lamp was utilized as the light source, and it was placed approximately 320 mm from the camera to ensure uniform lighting conditions [10]. An overview of the hyperspectral imaging system setup is shown in Fig. 3. To enable easy distinction between the epicuticular wax presence and absence in berries, 9 control berries were intentionally wiped with cotton cloths before imaging, creating a ‘No EW’ condition. A total of five spectral hypercubes were recorded: ‘No EW (9 images)’, ‘Perfect EW (10 images)’, ‘No EW vs. Perfect EW (10 images: 5 from No EW and 5 from Perfect EW)‘, ‘Assisted Harvested Blueberries (AHB: 10 images)’, and ‘Hand Harvested Blueberries (HHB: 10 images)’ (Fig. 4). shows an illustration of a false-colour image of the ‘No EW vs. Perfect EW’ captured with this NIR-HSI system. A brief overview of image structure, naming conventions, and data composition is provided in Table I.

**Fig. 5:**
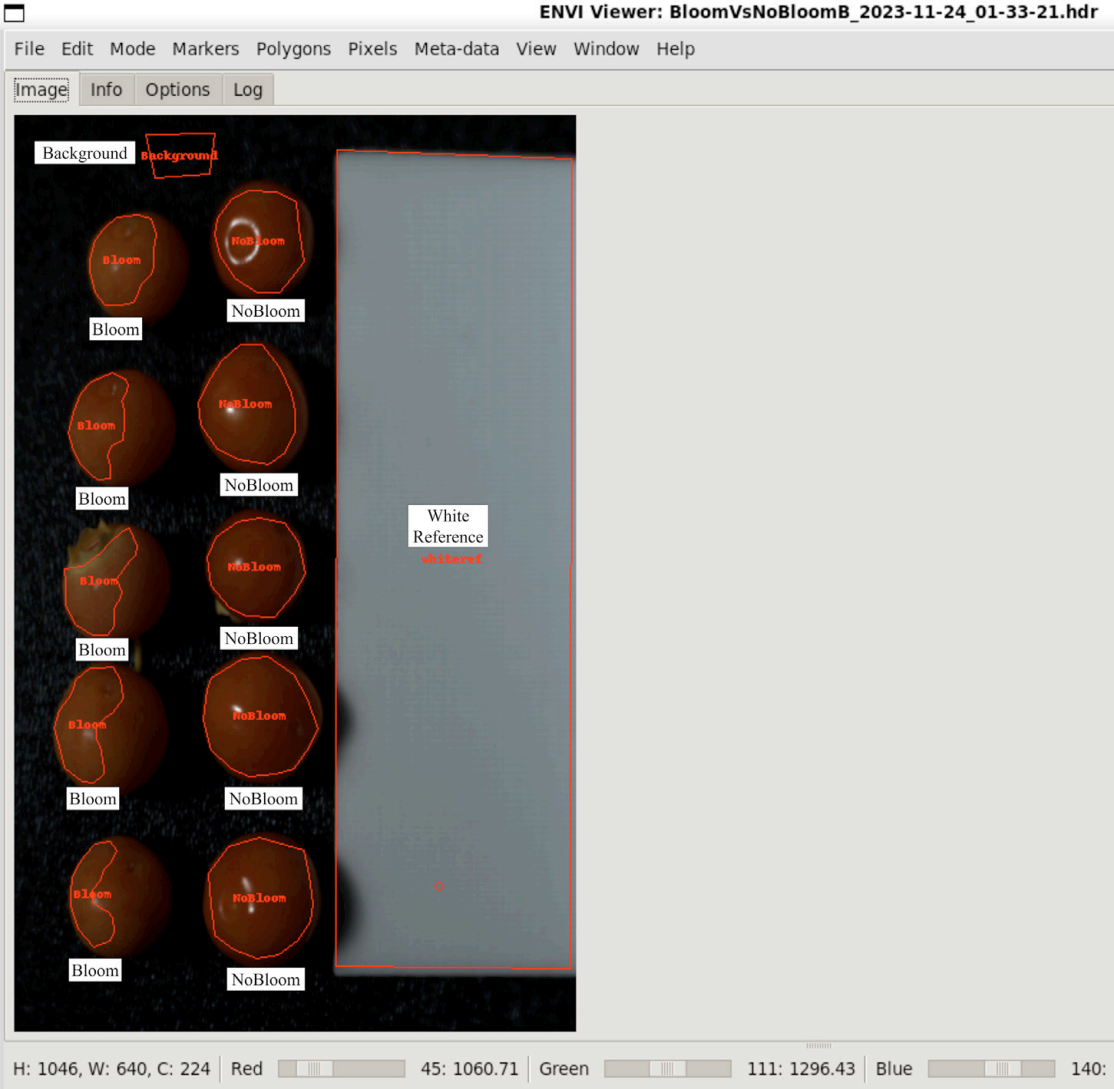
Illustration of the annotated image on the false-colour NIR image using HAPPy software [2].

## Limitations

This dataset has few constraints. One, there is small sample size, which could limit the statistical significance and generalizability. All blueberries were from a single cultivar (Masena) and sourced from one orchard due to resource limitations and proprietary access limitations. However, the samples were randomly selected in order to include overall quality evaluation of the blueberries. Future studies will focus on expanding the dataset to include samples from multiple orchards and different cultivars to increase the diversity and robustness. Moreover, the dataset is considered free from post-harvest damage, such as fungal infections or bruising, which is common in real-world applications and could have an impact on spectral signatures.

## Ethics Statement

Authors confirm that this dataset collection did not involve human subjects, animal experiment, or data from social media platforms.

## CRediT authorship contribution statement

**Shah Faisal:** Conceptualization, Methodology, Writing – original draft. **Yaminn Thawdar:** Data curation, Software. **Melanie Po-Leen Ooi:** Supervision, Conceptualization, Funding acquisition, Project administration, Writing – review & editing. **Peter Reutemann:** Formal analysis, Software, Resources. **Dale Fletcher:** Validation, Investigation. **Ye Chow Kuang:** Supervision, Conceptualization, Methodology, Validation. **Sanush K. Abeysekera:** Supervision, Writing – review & editing.

## Data Availability

DataverseBB-HSI: Proximal Hyperspectral Imaging Dataset for Masena Blueberries Epicuticular Wax Loss Identification and Post-Harvest Quality Evaluation. (Original data).
